# The Importance of High-Frequency Modes in the Prediction of RISC Rates for TADF Molecules

**DOI:** 10.1021/acs.jpclett.5c00176

**Published:** 2025-03-18

**Authors:** Teodoro Pizza, Amedeo Capobianco, Alessandro Troisi

**Affiliations:** † Dipartimento di Chimica e Biologia Adolfo Zambelli, Università di Salerno, Via Giovanni Paolo II, 132, I-84084, Fisciano, Salerno, Italy; ‡ Dipartimento di Chimica, Biologia e Biotecnologie, Università degli Studi di Perugia, Via Elce di Sotto, 8, I-06123, Perugia, Italy; § Department of Chemistry and Materials Innovation Factory, University of Liverpool, Liverpool, L69 7ZD, U.K.

## Abstract

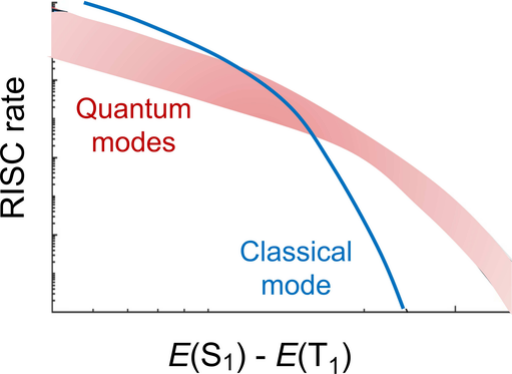

The reverse intersystem crossing (RISC) rate determines the efficiency of dyes displaying thermally activated delayed fluorescence (TADF). Such a rate can be predicted at the full quantum level by considering all the vibrational normal modes or adopting an approximated methodology which relies on single classical modes. We evaluated the importance of considering all of the vibrational degrees of freedom in computations for targeting the design of novel emissive materials from first principles. We computed the RISC rate for 17 molecules of interest for TADF by comparing a full quantum mechanics treatment based on Fermi’s golden rule with Marcus-based semiclassical approaches. The results are quantitatively and sometimes qualitatively different in the two approaches, especially when the reorganization energy is small, a common occurrence for molecules exhibiting TADF. The importance of high-frequency modes varies across the set of molecules considered, suggesting that their evaluation should become part of the molecular design process.

Computer-aided discovery of novel luminescent molecules is one of the most promising modern applications of quantum chemistry in materials science. It has been used, for example, to find novel acceptor/donor molecules for organic electronic applications,^1^ recognize new thermally activated delayed fluorescence (TADF) and singlet fission (SF) molecules,^2−4^ or to determine the suitability of organic semiconductor materials for technological applications.^5^ Many virtual screening approaches focus purely on energy alignment considerations, for example, to identify molecules with very close energy of first excited singlet and triplet states for TADF,^2^ with a specific frontier orbital energy level alignment for organic solar cells,^6,7^ or with the energy of their first excited singlet state being at least double the energy of the first excited triplet state for SF.^8^ This approach has been successful in some occasions,^2^ but it also led to consider molecules which did not display the desired properties, although exhibiting a favorable energy level alignment.^9^ Therefore, it is essential to employ a quantum chemical screening that also correctly accounts for the photophysical process of interest.

Considering, for example, the process of TADF, it is important to evaluate the rate of the reverse intersystem crossing (RISC) from the triplet state to the emissive singlet state, which is responsible for the delayed fluorescence. This rate, in turn, depends on the spectral density. The latter can be computed at the quantum mechanical level, by including all nuclear modes,^10^ or can be approximated, considering only classical modes.^11^ Evaluating the importance of including all of the nuclear modes in the computation of RISC rates is not a trivial task, as the rate also depends on the single-triplet energy gap and the spin–orbit coupling, both quantities being subject to experimental and computational uncertainties. There is also a range of methods which predict the global rate of the TADF process by means of kinetic models.^12,13^ Thus, determining the best approach to compute the rate is not only a very useful task but also clarifies whether the computation of the detailed vibronic structure of the nonradiative singlet–triplet transition should be included in the process of materials discovery.

In this work, we evaluate the importance of considering all normal modes in the computation of the RISC rate in search of novel TADF molecules. This will be done comparing a full quantum mechanical (QM) methodology developed on the framework of Fermi’s golden rule (FGR)^10^ with the semiclassical Marcus or Marcus-Levich-Jortner (MLJ) approaches^11,14^ for a sample of 17 molecules. We will consider only RISC rates from T_1_ to S_1_, even though we are aware of the possibility of having higher-energy triplet states involved in the TADF process.^14,15^ Furthermore, we do not explicitly consider effects due to aggregation in the solid state.^16^ However, accounting for aggregation and for the effect that T_2_, T_3_, or higher energy states can have on the final rate is not the main objective of this study and will not impact our conclusions. The molecules have been derived from refs (2, 14, and 17−21), and their structures are reported in Figure 1. Our choice was based on their energy gap, i.e., Δ*E*_ST_ = *E*_S_1__ – *E*_T_1__, if known experimentally, or on the predicted promising TADF behavior.^2,22^ This data set is representative of the TADF class, and the size is convenient to draw general conclusions, while allowing for the detailed inspection of individual entries.

**Figure 1:**
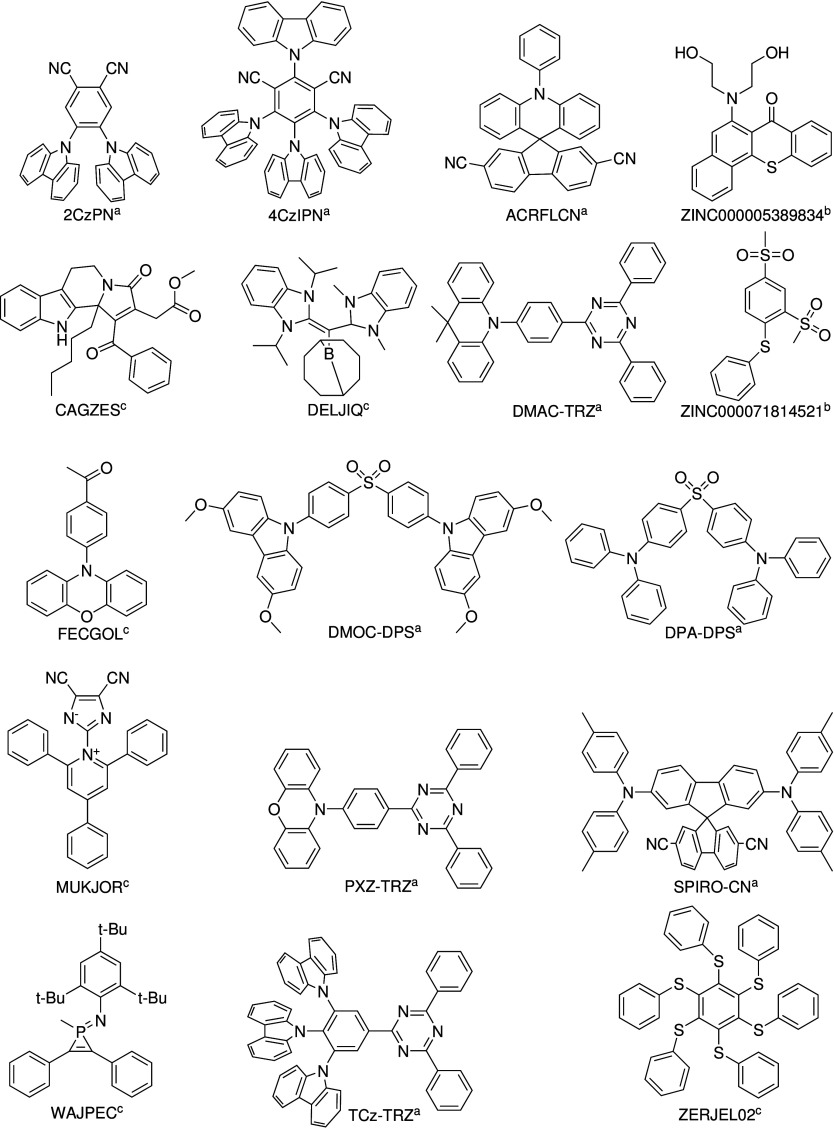
Molecules considered in this work with the label used in the experimental work^*a*^, ZINC identifier^*b*^, or CSD identifier^*c*^.

According to the principle of detailed balance, the RISC rate turns out to be
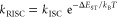
where *T* is temperature and *k*_B_ the Boltzmann constant. By using Fermi’s golden rule under the condition (herein met) that the energy gap is much larger than the spin–orbit coupling, |*H*_SO_|, assumed to be independent of the nuclear coordinates (Condon approximation), the intersystem crossing rate (*k*_ISC_) is evaluated as
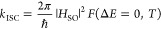
*F*(Δ*E*,*T*) is the Franck–Condon weighted density of states (FCWD):
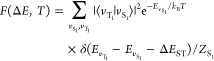
where Δ*E* is the argument of the delta function,  is the vibrational energy of T_1_(S_1_), *Z*_S_1__ the vibrational partition function of S_1_, and ⟨*v*_T_1__|*v*_S_1__⟩ the Franck–Condon integral. Upon introducing normal coordinates and Duschinsky affine transformation to express the set of normal coordinates of the initial electronic state as a function of those of the final state, the FCWD is computed via the generating function approach adopting the harmonic approximation for molecular vibrations.^10,23,24^ The affine transformation requires the computation of the Duschinsky matrix **J**, that takes into account mode mixing effects, and the displacement vector **K**, that accounts for the geometric displacement between the equilibrium geometries of the involved electronic states. It has already been shown that this approach can lead to reliable rates.^25−27^ Clearly, both optimized geometries and normal modes of the S_1_ and T_1_ states are needed to run these computations. We resorted to time-dependent density functional theory (TD-DFT) to treat the S_1_ state and ΔSCF for T_1_, employing the M06-2X functional and the 6-31G** basis set via the Gaussian 16 package.^28^ The polarizable continuum model (PCM) was employed to include bulk polarization. The relative permittivity was set to 4.0, which is a suitable value for mimicking the dielectric environment in organic solid-state devices.^29,30^ Spin–orbit couplings were computed with the ORCA software, by using the atomic mean-field approximation.^31^ FCWDs were computed by using a development version of the MolFC code.^32^ The internal representation of normal modes was used; see Section S1 in the Supporting Information for further details.^33^

Due to their inherent flexibility, molecules of interest for TADF often exhibit, predominantly at low frequencies, strongly anharmonic vibrational modes. While the best approach would be to explicitly consider the nonharmonic nature of these modes,^33^ in this study, we have opted for mode exclusion through the reduced dimensionality scheme—an algorithm initially proposed in refs^34, 35^. We implemented and validated an *ad hoc* version of this method, which ensures consistency across the data set and can be applied to multiple molecules automatically, without human intervention (see Section S1 and Figure S1 in the Supporting Information). Consequently, vibronic computations were performed on a subset of vibrational modes, termed active modes.

Mode exclusion is typically negligible for transitions involving the T_1_ and S_1_ states due to their low reorganization energies. Nevertheless, it becomes essential when calculating the FCWD for systems exhibiting a significant discrepancy between harmonic and TDDFT reorganization energy predictions, indicating a strong anharmonicity. We also considered the possibility of disregarding mode-mixing effects. However, ignoring mode-mixing occasionally leads to rate constants that differ by several orders of magnitude from those estimated by using the Duschinsky matrix and, in some cases, also to numerical instabilities in the autocorrelation function. See Table S1 in the Supporting Information.

Figure 2 shows a comparison between the observed and predicted FCWDs for the phosphorescence of four of the molecules considered in our study. Theoretical FCWDs show excellent agreement with their experimental counterparts, both in terms of band shape and bandwidth, further validating the effectiveness of our mode exclusion algorithm. It should be noted that (i) in predicted FCWD there is no additional bandwidth included by fitting and (ii) upon aligning the experimentally observed and the predicted FCWDs, the *E*_0–0_ energy of the transition can be inferred.^36^ See Figures S2–S10 and Table S2 in Section S2 of the Supporting Information.

**Figure 2:**
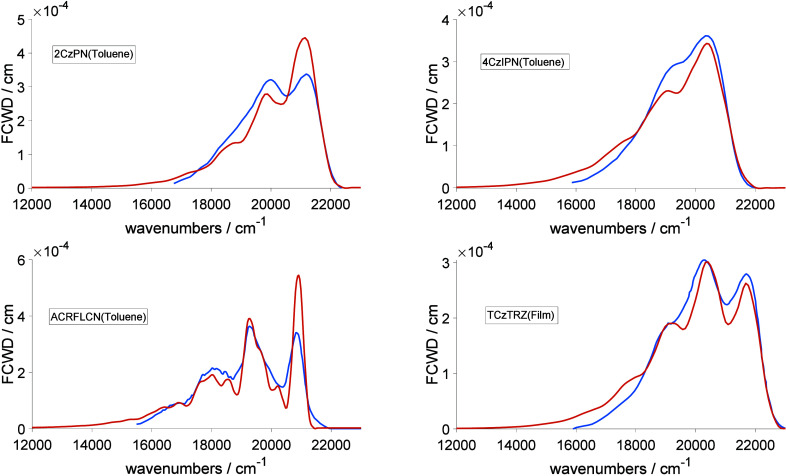
Superimposition of the experimental (blue line) and theoretical (orange line) FCWDs at *T* = 77 K, for the T_1_ → S_0_ phosphorescence of four of the molecules treated here: 2CzPN (top left), 4CzPN (top right), ACRFLCN (bottom left), and TCz-TRZ (bottom right). The experimental FCWDs have been digitized from refs (17, 18). Comparisons of predicted and observed FCWDs for fluorescence spectra are reported in the Supporting Information.

In ref (14), the MLJ approach was suggested as a viable tool for the computation of RISC rates. The authors showed that, for the systems studied, the inclusion of the effective high-frequency mode—evaluated using various values of reorganization energy, along with the effective Huang–Rhys factors and wavenumbers computed as in ref (37)—resulted in only minor variations (typically less than a factor of 2) in the predicted rates compared to those obtained using the Marcus equation with the same reorganization energy used in the MLJ equation. Consequently, they considered the limit of the negligible contribution from high-frequency modes, a choice that proved valid for the molecules analyzed in the original work. This also holds for the systems examined herein, as evidenced by the data in Table S3 of the Supporting Information, which confirm that incorporating the high-frequency mode has a negligible effect on the rates (again, less than a factor of 2) and demonstrate that the Marcus equation provides a reliable approximation of the MLJ results within a Δ*E*_ST_ range amounting to 0.5 eV for the systems herein investigated.

To highlight the role of a full quantum mechanics treatment of FCWDs we report in Figure 3 the rates as a function of Δ*E*_ST_ scaled by the prefactor |*H*_SO_|^2^. In this way our results are independent of the accuracy in the determination of Δ*E*_ST_ and *H*_SO_, which are also highly important, but not the focus of this study.^38^

**Figure 3:**
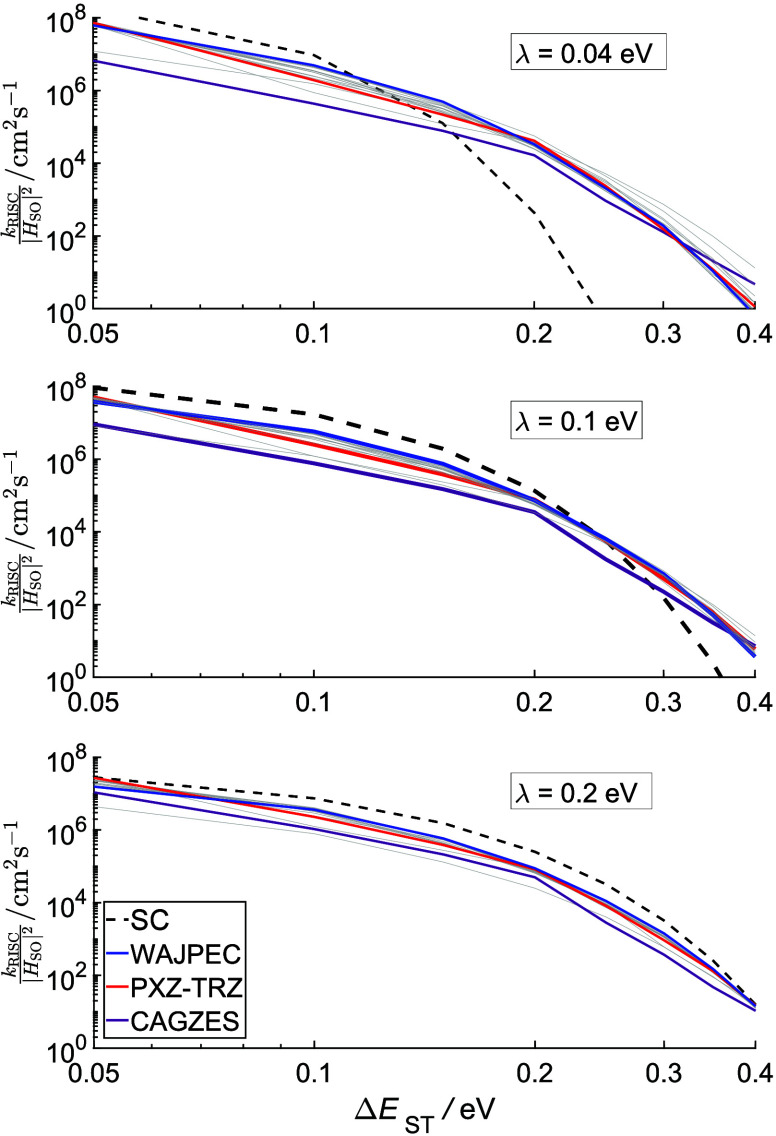
Relative RISC rate constant (*k*_RISC_/|*H*_SO_|^2^) as a function of Δ*E*_ST_ at *T* = 300 K. The SC curve (black dashed line) has been computed by using the Marcus equation as in ref (14). The other curves have been obtained with our full quantum mechanics treatment. Three different reorganization energies (λ) have been considered: 0.04 eV (top panel), 0.1 eV (middle panel), and 0.2 eV (bottom panel). Three systems: WAJPEC, PXZ-TRZ, and CAGZES are put into evidence by blue, red, and purple lines, respectively.

Although the Marcus equation has been used for the dashed lines of Figure 3 it should be emphasized that the analysis of those results can be safely extended to the MLJ approach.

QM rates have been computed by rescaling the displacement vector **K** in order to have the same reorganization energy (e.g., λ = 0.1 eV, Figure 3, middle panel) for all molecules. That choice is aimed at isolating the effect of accurate calculation of FCWD from other molecule-specific parameters, thus enabling a detailed understanding of its role. The range of Δ*E*_ST_ used in Figure 3 reflects the experimental range.

At small Δ*E*_ST_, the range of molecules considered here displays a computed RISC rate about 1 order of magnitude smaller than predictions based on semiclassical (SC) models, with a significant molecule-to-molecule difference which is obviously missed by the SC approach. We took as a case study the rates at Δ*E*_ST_ = 0.1 eV of the highlighted molecules (blue, red, and purple lines, Figure 3). For these molecules, at *T* = 300 K, SC predicts a RISC rate of 1.7 × 10^7^ cm^2^s^–1^, which would make a good TADF. However, accounting for a full quantum mechanics FCWD results in only some molecules achieving that outcome. As a matter of fact, the WAJPEC-like molecule seems to be a good TADF candidate, having a rate close to the one obtained by SC predictions (*k*_RISC_/|*H*_SO_|^2^ = 5.8 × 10^6^ cm^2^s^–1^, blue curve); a lower rate is predicted at the QM level for PXZ-Trz (*k*_RISC_/|*H*_SO_|^2^ = 2.5 × 10^6^ cm^2^s^–1^, red curve), whereas the CAGZES-like molecule seems to behave markedly worse as a TADF, its rate (*k*_RISC_/|*H*_SO_|^2^ = 7.6 × 10^5^ cm^2^s^–1^, purple curve) being much lower than the one obtained with the SC approach. Notwithstanding the better agreement at higher Δ*E*_ST_, the majority of known efficient TADF molecules show very small Δ*E*_ST_ (See Table S2 in the Supporting Information), usually lower than 0.2 eV, which is the region where the disagreement between the two methods seems to be more important. Let us examine another representative case: λ = 0.04 eV, which is the median of the classical part of the reorganization energies of the molecules studied here. As seen from Figure 3 (top panel), the agreement between the two models seems to be somewhat better in the region going from 0.1 to 0.15 eV. Instead, outside of this range, the gap between the models is so large that using the SC approach to compute RISC rates could lead to a substantial error in the interpretation of the results for some of them. A better agreement between the QM and SC approaches is found at higher λ, e.g., λ = 0.2 eV, a value not unusual for organic molecules but larger than the median computed from our representative sample (Figure 3, bottom panel).

The results in Figure 3 are further validated by the RISC rates calculated using both the QM and MLJ models, where the spin–orbit coupling and reorganization energies are predicted by M06-2X computations, in combination with the experimentally observed singlet–triplet energy differences (for 9 out of 17 cases where such data are available) or calculated values. Predicted rate ratios, diagrammed in Figure S11 (see also Tables S1 and S4 of the Supporting Information), reveal that the largest discrepancies (several orders of magnitude) between the rates predicted by the MLJ equation and the QM approach occur for the systems with low reorganization energy (λ ≤ 0.1 eV), which is the most common case for TADF, with the differences being more relevant for larger values of the singlet–triplet energy gap, see Figure S12 of the Supporting Information. We also conducted a fitting procedure to determine the hypothetical rescaling of the classical component of the reorganization energy able to reproduce the experimental (where available) or QM-predicted rates using the MLJ equation in conjunction with the Huang–Rhys factor and the wavenumber of the effective mode of the quantum component computed according to well-established procedures.^37^ This test (see Table S4 of Supporting Information) shows that the optimal reorganization energy to be included in the MLJ equation in fact exceeds the total reorganization energy obtained from TDDFT computations for all of the investigated systems, being on average six times larger than the total reorganization energy. This further suggests that semiclassical approaches may present limitations as purely predictive tools, limited to the cases investigated herein.

A substantial deviation of the QM from the MLJ model can be expected when high-frequency (i.e., less classical) modes are involved in the nonradiative channel. This is confirmed by the inspection of Figure 4 where per-mode reorganization energies are plotted against wavenumbers for three molecules going from the better (top, blue) to the worse (bottom, purple) accordance between the QM and MLJ approaches for λ = 0.1 eV and Δ*E*_ST_ = 0.1 eV (Figure 3, middle panel).

**Figure 4:**
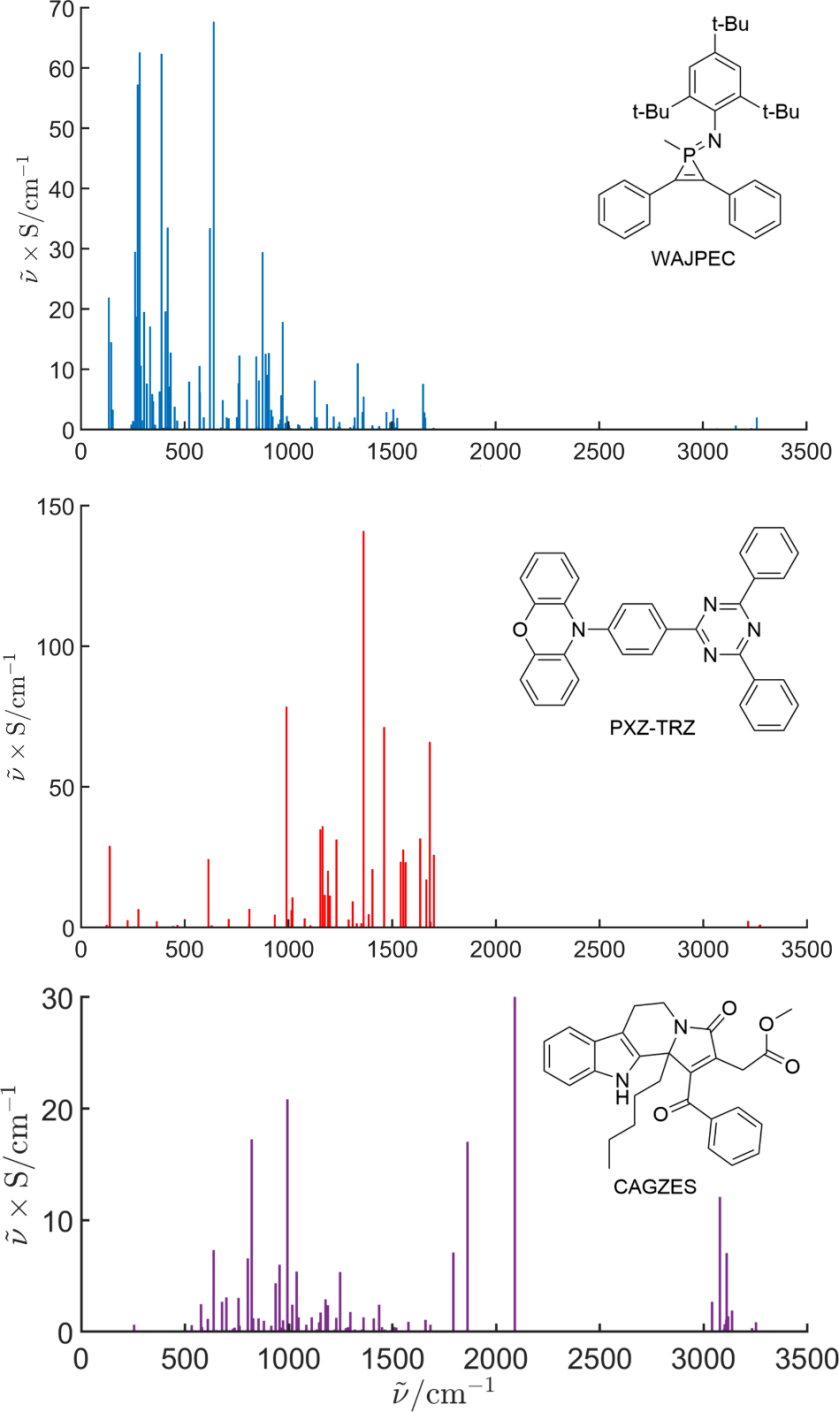
Per-mode reorganization energies (ν̃ × S, where *S* is the Huang–Rhys factor) as a function of wavenumbers . Three molecules are considered: WAJPEC (top panel), PXZ-TRZ (middle panel), CAGZES (bottom panel).

However, performing a visual inspection of per-mode reorganization energy may become unfeasible when dealing with a larger sample of molecules. For this reason, the parameter of the MLJ equation that accounts for all contributing modes, i.e., the effective mode wavenumber, , can be used to visualize this trend:
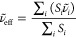
1where *S*_*i*_ is the Huang–Rhys factor of the *i*-th mode and  its wavenumber. Having removed low-frequency modes in the computation of FCWD (*vide supra*), this parameter closely resembles the one used in ref (14) where  was employed as the wavenumber of the effective mode for the up-conversion process. The vibrational progression ensuring the nonradiative channel is mainly ascribable to low-frequency modes for lower  or high-frequency modes for higher . For the molecules taken into consideration (WAJPEC, PXZ-TRZ, and CAGZES, Figure 4),  assumes the values of 410, 927, and 1747 cm^–1^. In accordance with previous considerations (compare blue, red, and purple curves with the black-dashed one in Figure 3), the discrepancy between the SC and QM prediction increases upon increasing , thus further demonstrating the value of the QM approach when high-frequency modes play a key role in the RISC process.

We studied a sample of 17 organic molecules, known or proposed to display TADF, to evaluate the importance of including their nuclear modes quantum mechanically in the evaluation of the RISC rate. To isolate this specific effect, we considered suitably rescaled rates where the only difference is in the treatment of the nuclear modes regardless of the specific value of the spin–orbit coupling, reorganization energy, and energy gap. An *ad hoc* algorithm has been introduced to remove anharmonic low-frequency modes in a consistent way across the data set and enables the potential deployment of this methodology on a larger number of molecules. We find that the inclusion of quantum nuclear modes is essential for low reorganization energies and a low singlet–triplet gap, i.e., precisely where the TADF phenomenon is more likely. Besides, the MLJ approach is not fully reliable in predicting molecule-to-molecule differences caused by the different contribution of high-frequency vibrational modes. Therefore, considering all normal modes at a quantum level becomes crucial in order to avoid misidentification of potential TADF candidates. Interestingly, the inclusion of all the active normal modes causes a computational cost increase of only 30% (due to the computation of normal modes of the S_1_ state). In the context of virtual screening, where the photophysical rates are computed only for the candidates that are most promising on the basis of energy-level alignment, this represents a very marginal increase of the overall cost of virtual screening; therefore, the computation of the FCWD at the quantum level is worth considering for the sake of accuracy.

## Supporting Information Available

Validation of the method used to remove normal modes (Figure S1); Δ*E*_ST_ energy gap, reorganization energies and RISC rates predicted by the QM model (Table S1); predicted 0–0 transition energies, as inferred from the overlap of experimental and theoretical fluorescence FCWDs, and comparison with the experimental values (Figures S2–S10, Table S2); RISC rates predicted by the Marcus and the MLJ equations (Table S3); spin–orbit couplings, Huang–Rhys factors, effective wavenumbers and reorganization energies used in MLJ equation (Table S4); RISC rates calculated at the QM and MLJ levels by using the spin–orbit coupling and the reorganization energy as predicted by M06–2X computations, in conjunction with the experimentally observed or calculated singlet–triplet energy differences as a function of the reorganization energy (Figure S11) and the energy gap (Figure S12) (PDF)
