# Supplementary material for: The Importance of High-Frequency Modes in the Prediction of RISC Rates for TADF Molecules
Source: J Phys Chem Lett. 2025 Mar 18;16(12):3056–62. doi: 10.1021/acs.jpclett.5c00176 (PMC11956131; doi:10.1021/acs.jpclett.5c00176)
Supplement: Supplementary file 1 — jz5c00176_si_001.pdf [file jz5c00176_si_001.pdf]

Supporting Information: The Importance of High Frequency Modes in the Prediction of RISC Rates for TADF Molecules

Teodoro Pizza,[†,‡] Amedeo Capobianco,[*,†] and Alessandro Troisi[*,¶]

†*Dipartimento di Chimica e Biologia "A. Zambelli", Università di Salerno, Via Giovanni Paolo II, 132, I-84084 Fisciano (SA), Italy*

‡*Dipartimento di Chimica, Biologia e Biotecnologie, Università degli Studi di Perugia, Via Elce di Sotto, 8, I-06123 Perugia (PG), Italy*

¶*Department of Chemistry and Materials Innovation Factory, University of Liverpool, Liverpool, L69 7ZD, UK*

E-mail: acapobianco@unisa.it; a.troisi@liverpool.ac.uk

Contents

S1. Reduced dimensionality scheme **S-2**

S2. E_{0-0} energy by the alignment of observed and predicted FCWD **S-6**

S3. RISC rates by the MLJ and Marcus equations **S-13**

S1. Reduced dimensionality scheme

In our implementation of the generating function, the computation of the Franck-Condon weighted density of states (FCWD) adopts the Duschinsky affine transformation to relate the normal coordinates of the final state (B) to the ones of the initial state (A):

$$\mathbf{Q}_{\mathrm{B}} = \mathbf{J}\mathbf{Q}_{\mathrm{A}} + \mathbf{K}, \tag{1}$$

in which $\mathbf{Q}$ are normal coordinates, $\mathbf{J}$ is the Duschinsky matrix that takes into account mode mixing effects and $\mathbf{K}$ is the shift vector that accounts for the geometric displacement between the equilibrium geometries of the involved electronic states. Although the internal coordinate representation of the normal modes (herein adopted) usually mitigates the unrealistic shifts of stretching coordinates caused by the displacement of angular coordinates upon the geometry change between initial and final state,[S1] the computation of the FCWD can be still very challenging for large-size flexible systems. Indeed, those molecules exhibit large amplitude modes for which the harmonic approximation, on which the generating function approach is based, is very poor. Fortunately, in most cases, as shown in refs S2 and S3, for such flexible molecules a reduced dimensionality scheme can be applied. That method, whose nature is totally empirical, is able to identify and remove the normal modes that cause unphysical progressions in the FCWD. In detail, the space of vibrational degrees of freedom is reduced, by leaving only the so-called active modes, *i.e.* the modes that – hopefully – really contribute to the FCWD.

We have implemented and introduced in our methodology an *ad hoc* version of that scheme which herein depends on two adjustable parameters to be optimized for the specific class of investigated molecules: A threshold wavenumber ($\tilde{\nu}_{\mathrm{t}}$) and a positive number η, whose value cannot exceed 1. At first, only the vibrational degrees of freedom with harmonic wavenumbers higher than $\tilde{\nu}_{\mathrm{t}}$ are retained, while the remaining ones are removed from the vibrational spaces. Then, for both electronic states, the protocol attempts to find (and save)

the normal modes which are scarcely coupled with the remaining ones. The total coupling, C_j of the j-th mode is determined by summing the squares of the elements of the Duschinsky matrix J_{kj}:

$$C_j = \sum_{k=1}^{N_{\text{red}}} {J_{kj}}^2 / S_j,$$

where, at the first step, N_{red} is the number of vibrational modes left after the initial screening on $\tilde{\nu}_{\text{t}}$; S_j is a normalization factor that takes into account the non orthogonality of $\mathbf{J}$ and is defined as

$$S_j = \sum_{k=1}^{N_{\text{vib}}} {J_{kj}}^2,$$

in which N_{vib} is the total number of vibrational degrees.

If $C_j \geq \eta$, the j-th mode is retained, otherwise it is removed. The process is repeated iteratively and C_j is updated by summing ${J_{kj}}^2/S_j$ on the progressively reduced space until no more modes are removed both in the initial and the final state. For the molecules investigated here, we found $\tilde{\nu}_{\text{t}} = 100$ cm^{-1} and $\eta = 0.9$ as the optimized parameters. Figure S1 demonstrates the improvement of the agreement between the theoretical and experimental FCWDs when we introduce this algorithm for one of the molecules considered in this work. Table S1 reports RISC rates predicted by QM computations with mode exclusion, without mode exclusion and without including Duschinsky mode-mixing.

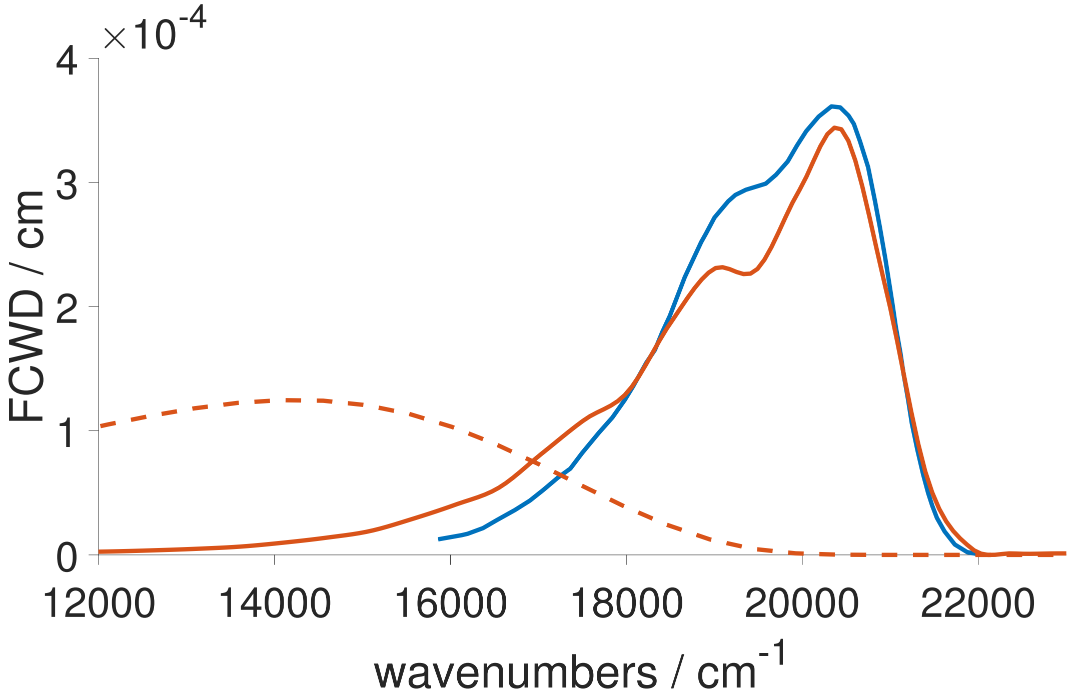

Figure S1: Experimental (blue) and theoretical (orange) FCWDs for the phosphorescence of 4CzIPN at $T = 77$ K in toluene. Full(dashed) orange line: predicted FCWD with(without) application of the reduced dimensionality scheme. Without reducing the vibrational space (dashed line), predicted FCWD is extremely broadened due to the presence of highly anharmonic modes, which leads to unwanted results.

Table S1: Singlet-triplet energy gap (ΔE_{ST}, eV);[a] reorganization energies (λ, eV) predicted by the harmonic model and by TDDFT computations; RISC rates (s^{-1}) predicted by QM computations with mode exclusion ($k^{\mathrm{e}}_{\mathrm{QM}}$), without mode exclusion ($k^{\mathrm{ne}}_{\mathrm{QM}}$) and without including Duschinsky mode-mixing ($k^{\mathrm{nd}}_{\mathrm{QM}}$).

Molecule ID	ΔE_{ST}[a]	λ^{harm}	λ^{TDDFT}	$k^{\mathrm{e}}_{\mathrm{QM}}$	$k^{\mathrm{ne}}_{\mathrm{QM}}$	$k^{\mathrm{nd}}_{\mathrm{QM}}$
PXZ-TRZ	*0.06*[e]	0.01	0.01	7.45×10^{4}	1.81×10^{4}	3.89×10^{4}
4CzIPN[b]	*0.10*[e]	0.09	0.08	1.86×10^{6}	1.54×10^{5}	4.60×10^{5}
ACRFLCN[c]	*0.03*[f]	0.004	0.004	2.62×10^{7}	2.35×10^{7}	2.54×10^{7}
DMAC-TRZ	*0.05*[g]	0.04	0.03	1.16×10^{7}	1.01×10^{6}	5.81×10^{6}
DMOC-DPS	*0.24*[h]	0.03	0.06	2.20×10^{2}	1.01×10^{3}*	1.96×10^{3}*
DPA-DPS	*0.52*[e]	0.08	0.10	1.67×10^{-4}	1.50×10^{-3}	1.39×10^{-4}
2CzPN[d]	*0.31*[e]	0.68	0.21	5.50×10^{3}	5.30*	5.20×10^{2}*
SPIRO-CN	*0.06*[e]	0.003	0.003	1.22×10^{5}	1.63×10^{5}	1.81×10^{5}
TCz-TRZ	*0.12*[i]	0.08	0.08	2.33×10^{5}	1.02×10^{5}	3.33×10^{5}
WAJPEC	0.51	0.07	0.02	2.70×10^{-4}	5.07×10^{-2}	1.22×10^{-4}
CAGZES	0.51	1.14	0.17	2.41×10^{-2}	4.70×10^{-3}*	3.93×10^{-2}
DELJIQ	0.29	0.35	0.21	4.42×10	4.80×10	2.22×10^{2}
FECGOL	0.37	0.03	0.07	9.30×10^{-2}	4.02*	4.28*
MUKJOR	0.59	1.06	0.18	2.45×10^{-4}	1.54×10^{-4}	8.61×10^{-5}
ZINC000005389834	0.37	0.03	0.02	1.71×10^{-1}	5.23×10^{-1}	6.44×10^{-2}
ZINC000071814521	0.25	0.78	0.46	3.74×10^{5}	1.47×10^{5}	9.93×10^{4}
ZERJEL02	0.71	8.72	0.28	1.40×10^{-3}	9.28×10^{-7}*	9.88×10^{-20}*

[a]Singlet-triplet energy gaps predicted at the (TD)M06-2X/6-31G** level, but for the first nine entries (italicized), where experimentally determined values were employed. [b]The experimental value of k_{RISC} is 2.7×10^{6} s^{-1} (ref. S4). [c]The experimental value of k_{RISC} is 2.5×10^{7} s^{-1} evaluated by using k_{ISC} and ΔE_{ST} given in ref. S5. [d]The experimental value of k_{RISC} is 5.4×10^{3} s^{-1} (ref. S4). The * symbol indicates cases where the FCWD exhibits numerical instabilities, which were found only when mode removal was not applied or when Duschinsky effects were not considered. [e]Ref S6. [f]Ref S5. [g]Ref S7. [h]Ref S8. [i]Ref S9.

S2. E_{0-0} energy by the alignment of observed and predicted FCWD

As is well known, for the radiative emission A $\rightarrow$ B, where A and B are electronic states, upon introducing the Born-Oppenheimer and the Condon approximations, the FCWD turns out to be:

$$F(hc\tilde{\nu}, T) = \sum_{a,b} |\langle b|a\rangle|^2 \, \mathrm{e}^{-\beta E_a} \delta \left(E_B - E_A + E_b - E_a + hc\tilde{\nu}\right)/Z_a,$$

where $\tilde{\nu}$ is the wavenumber of the radiation, $\beta = (k_\mathrm{B}T)^{-1}$, with T temperature and k_B the Boltzmann constant. $E_{A(B)}$, is the electronic energy, $E_{a(b)}$ the vibrational energy of A(B), Z_a is the vibrational partition function of the initial state and $\langle b|a\rangle$ is the Franck-Condon integral. Because we put $E_B - E_A = 0$ in the computation of the FCWD and because the FCWD must be normalized in the sense of unit area, upon aligning the predicted FCWD with the one extracted from the observed spectrum (and kept fixed) in such a way to maximize the overlap between the curves, it is possible to locate the 0-0 transition energy, see Figures S2-S10 and Table S2.

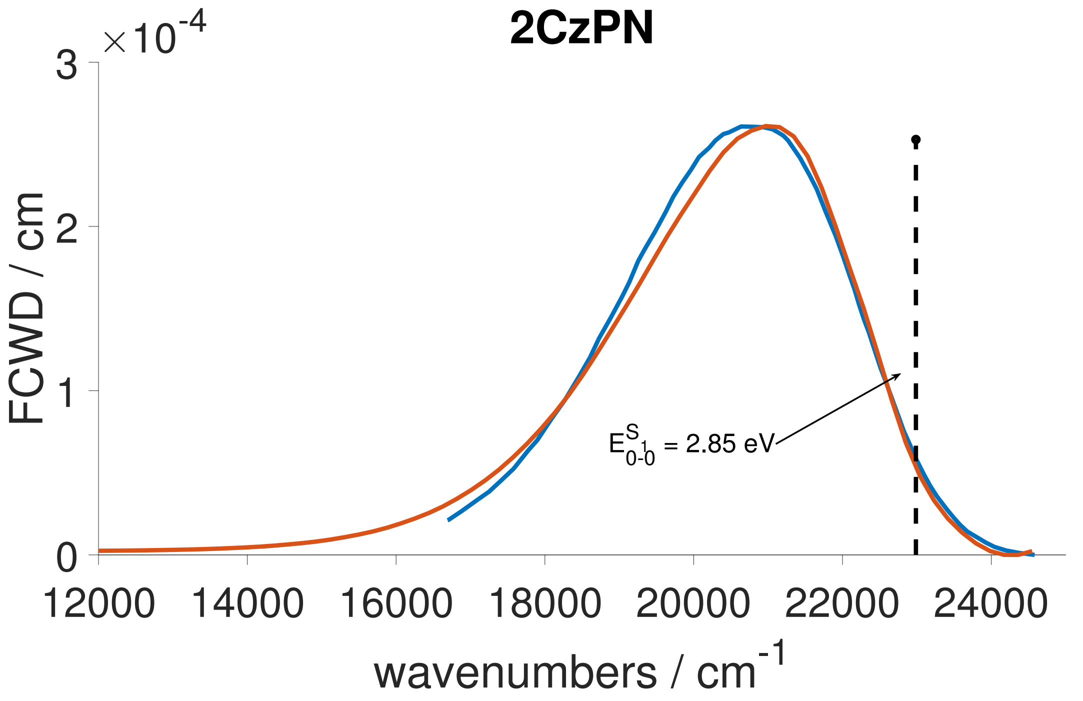

Figure S2: Experimental (blue) and theoretical (orange) FCWDs for the fluorescence of 2CzPN at $T = 300$ K in toluene. The 0-0 transition energy is indicated by the dashed black line. See Table S2 for a comparison with the experimental value.

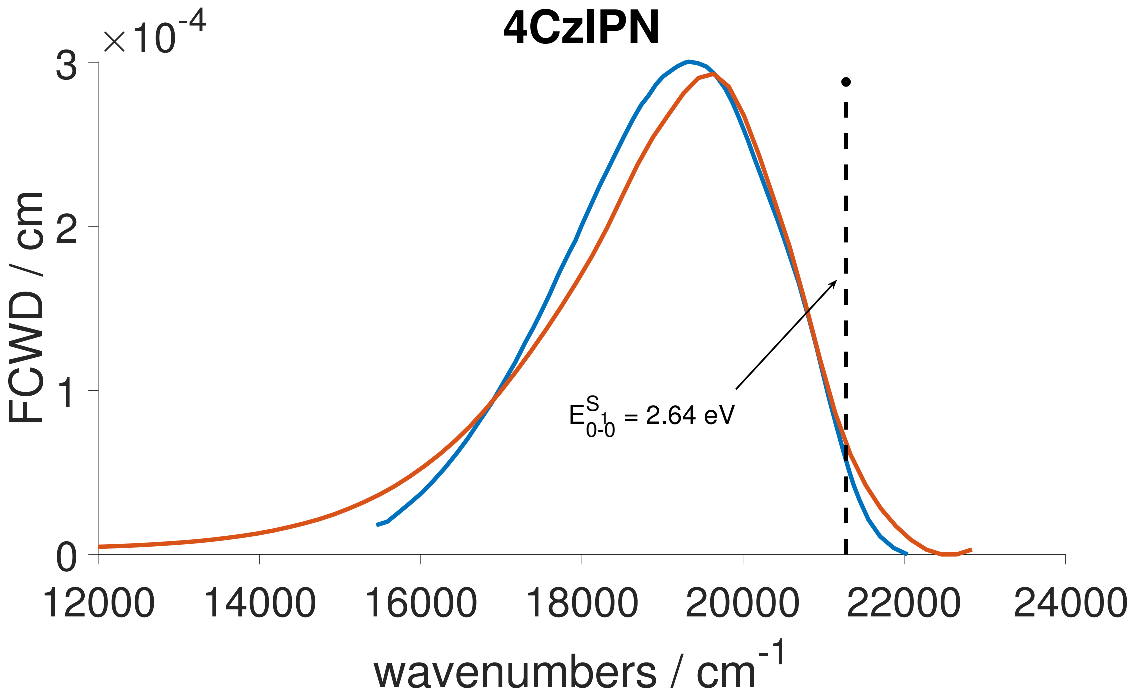

Figure S3: Experimental (blue) and theoretical (orange) FCWDs for the fluorescence of 4CzIPN at $T = 300$ K in toluene. The 0-0 transition energy is indicated by the dashed black line. See Table S2 for a comparison with the experimental value.

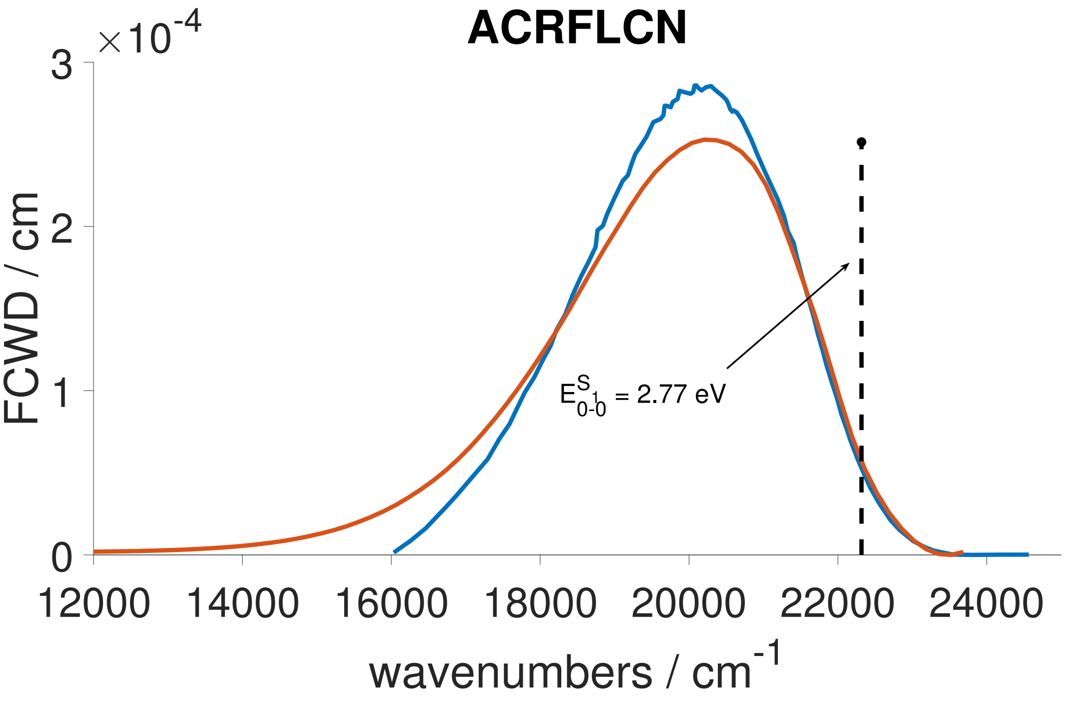

Figure S4: Experimental (blue) and theoretical (orange) FCWDs for the fluorescence of ACRFLCN at $T = 300$ K in toluene. The 0-0 transition energy is indicated by the dashed black line. See Table S2 for a comparison with the experimental value.

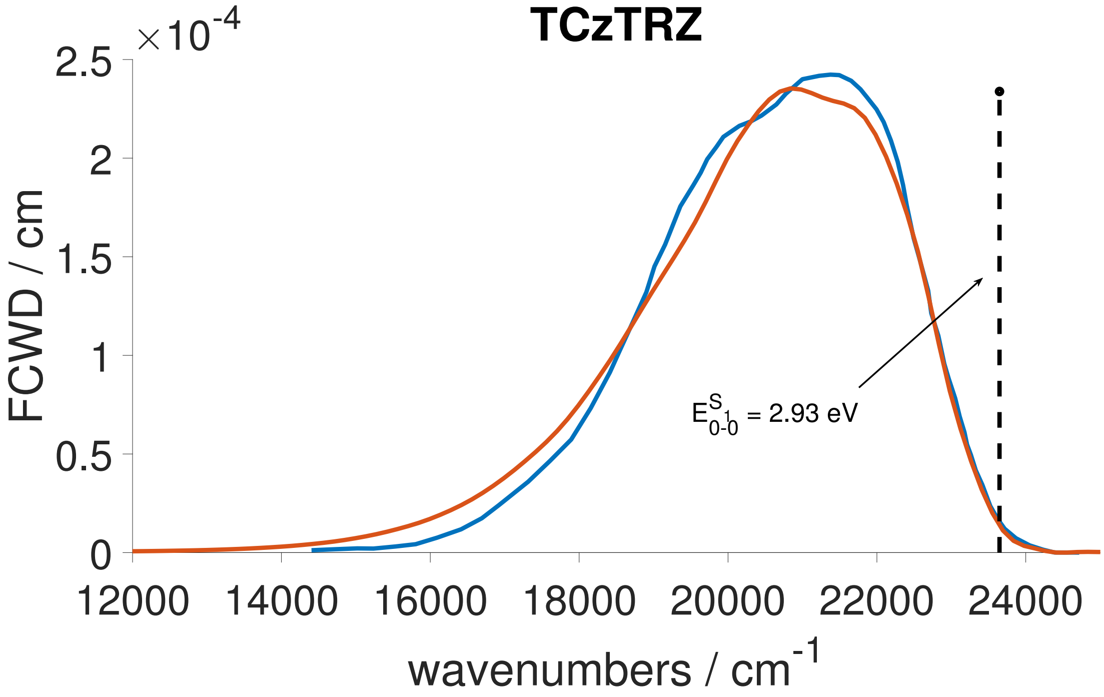

Figure S5: Experimental (blue) and theoretical (orange) FCWDs for the fluorescence of TCzTRZ at $T = 300$ K in a 6% doped DPEPO host film. The 0-0 transition energy is indicated by the dashed black line. See Table S2 for a comparison with the experimental value.

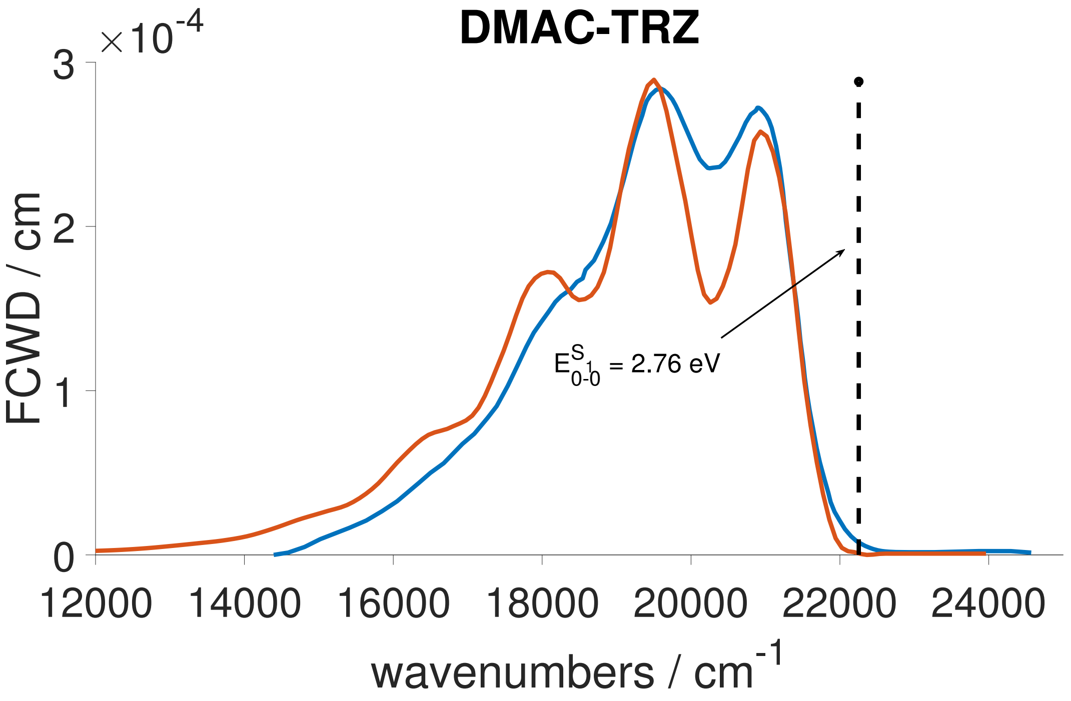

Figure S6: Experimental (blue) and theoretical (orange) FCWDs for the fluorescence of DMAC-Trz at $T = 300$ K in hexane. The 0-0 transition energy is indicated by the dashed black line. To the best of our knowledge, no experimental value could be found in the literature.

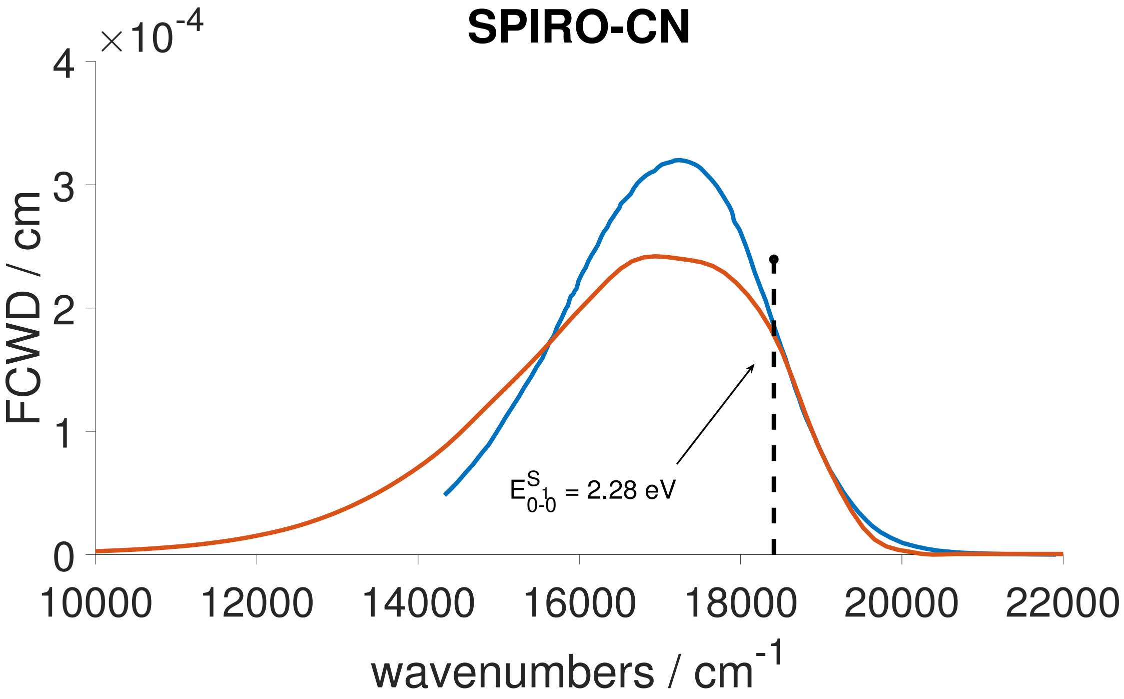

Figure S7: Experimental (blue) and theoretical (orange) FCWDs for the fluorescence of SPIRO-CN at $T = 300$ K in toluene. The 0-0 transition energy is indicated by the dashed black line. See Table S2 for a comparison with the experimental value.

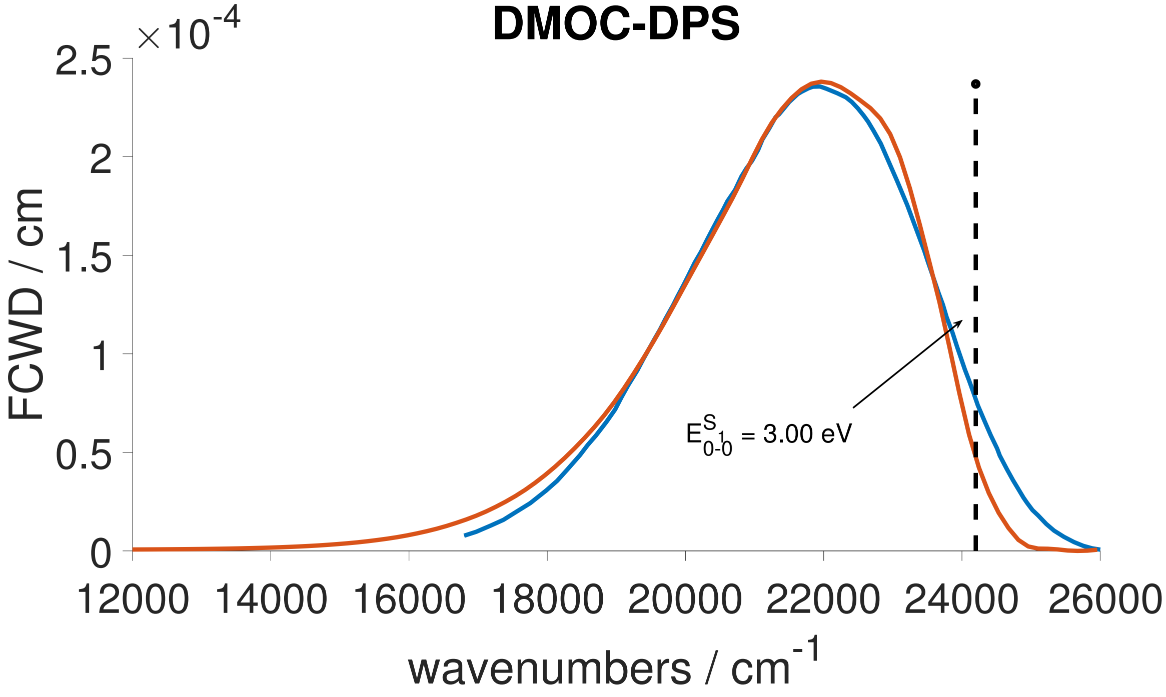

Figure S8: Experimental (blue) and theoretical (orange) FCWDs for the fluorescence of DMOC-DPS at $T = 300$ K in toluene. The 0-0 transition energy is indicated by the dashed black line. See Table S2 for a comparison with the experimental value.

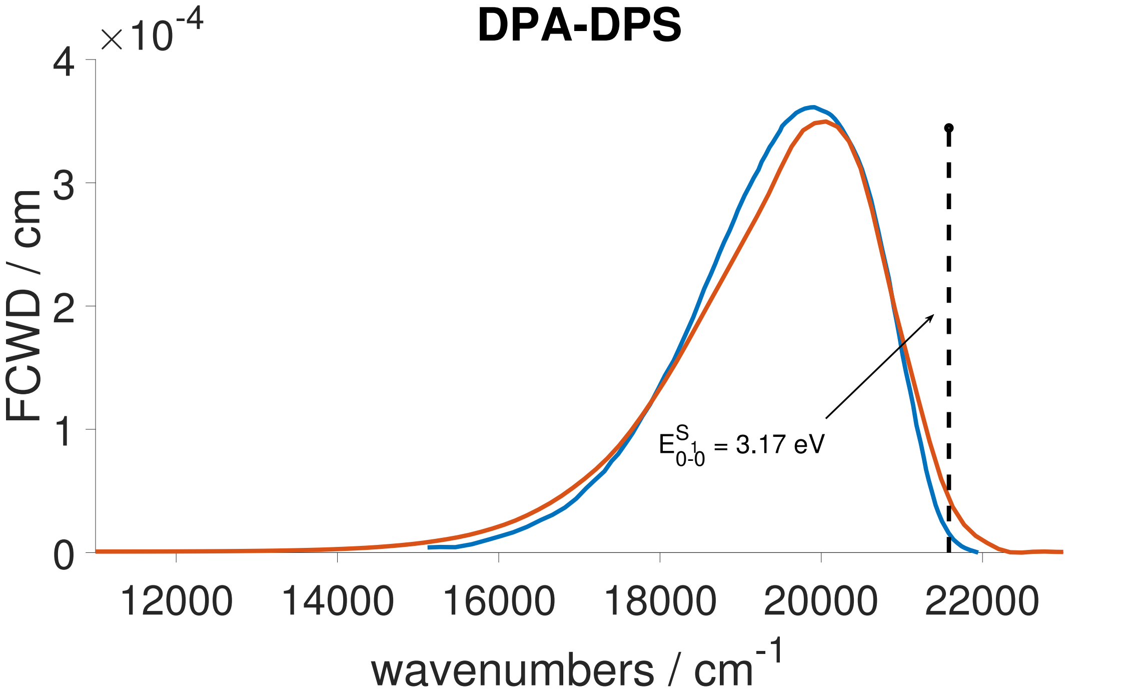

Figure S9: Experimental (blue) and theoretical (orange) FCWDs for the fluorescence of DPA-DPS at 300 K in toluene. The 0-0 transition energy is indicated by the dashed black line. See Table S2 for a comparison with the experimental value.

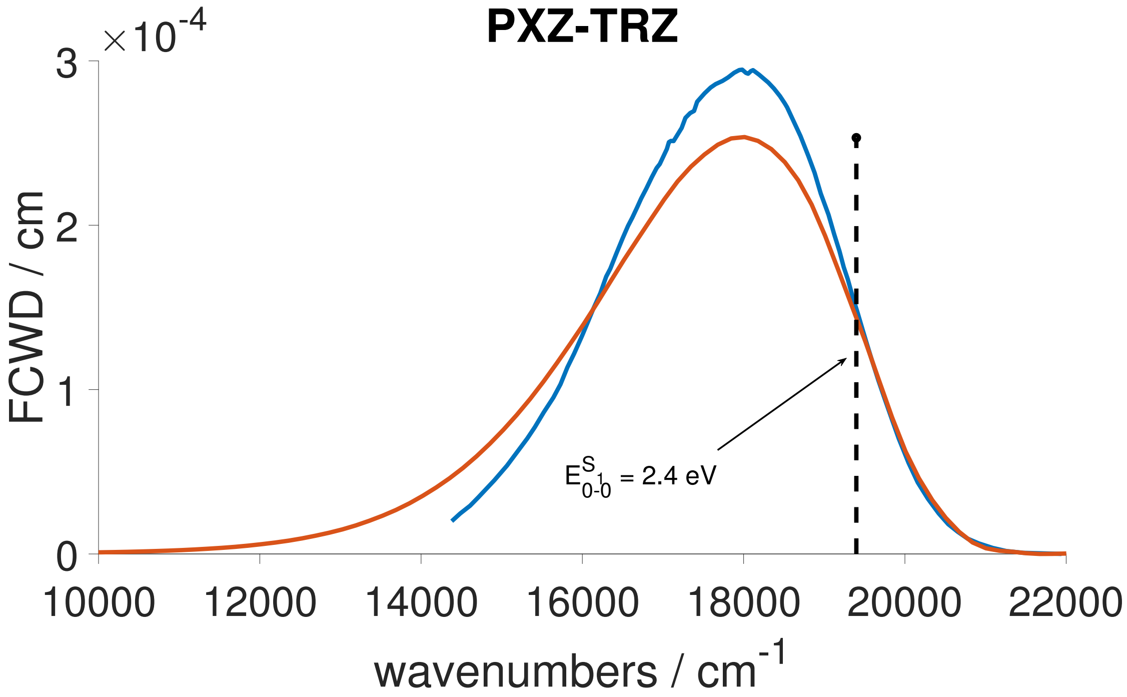

Figure S10: Experimental (blue) and theoretical (orange) FCWDs for the fluorescence of PXZ-Trz at $T = 300$ K in toluene. The 0-0 transition energy is indicated by the dashed black line. See Table S2 for a comparison with the experimental value.

Table S2: Observed and predicted E_{0-0} energy (eV) for the $S_1 \rightarrow S_0$ emission for the system whose E_{0-0} energy was already reported in the literature. Predicted values have been obtained by superimposing observed and computed FCWDs see Figures S2-S10. Experimental energies were taken from ref S6 unless otherwise noted. All the experimental spectra were measured in toluene solution, but for TCzTRZ, whose fluorescence was observed in a 6% wt doped DPEPO host film. The polarizable continuum model (PCM) was used to include solvent polarization in computations. Toluene was adopted as the solvent, but for TCzTRZ, for which the relative permittivity was set to 4.0 in PCM computations (see main article).

Molecule ID	$E^{S_1}_{0-0,\text{exper.}}$	$E^{S_1}_{0-0,\text{theor.}}$
4CzIPN	2.63	2.64
2CzPN	2.94	2.85
ACRFLCN[a]	2.61	2.75
PXZ-TRZ	2.53	2.42
DPA-DPS	3.28	3.18
SPIRO-CN	2.44	2.28
TCzTRZ[b]	2.94	2.93
DMOC-DPS[c]	3.12	3.00

[a]Experimental E_{0-0} energy from ref S5. [b]Experimental E_{0-0} energy from ref S9. [c]Experimental E_{0-0} energy from ref S8.

S3. RISC rates by the MLJ and Marcus equations

In the framework of Marcus-Levich-Jortner (MLJ) theory, the total reorganization energy (λ) is divided into two components: the low-frequency reorganization energy, λ_{S}, which accounts for the reorganization due to low-frequency vibrational modes and the surroundings, and the high-frequency contribution, which is associated with high-frequency intramolecular vibrational modes. The latter term is modeled using an effective vibrational mode characterized by an effective Huang-Rhys factor, S_{eff}, and an effective wavenumber, $\tilde{\nu}_{\mathrm{eff}}$.[S10]

In the harmonic approximation, the Huang-Rhys factor S_i for a vibrational mode i is a dimensionless quantity given by:

$$S_i = \frac{\lambda_i}{\tilde{\nu}_i}, \tag{2}$$

where $\tilde{\nu}_i$ is the wavenumber of the i-th mode, and λ_i is its reorganization energy, which, when expressed as a wavenumber, is given by:

$$\lambda_i = \frac{2\pi^2 c \tilde{\nu}_i^2 (\Delta Q_i)^2}{h}, \tag{3}$$

where ΔQ_i, with Q_i being a mass-weighted normal coordinate, represents the contribution of the i-th mode to the geometric change between the equilibrium geometries of the involved states. If normal coordinates are adopted in conjunction with the Duschinsky transformation, then ΔQ_i corresponds to the i-th element of the $\mathbf{K}$ vector in Eq 1.

If S_i is the Huang-Rhys factor for the i-th vibrational mode, and its wavenumber satisfies the condition $\tilde{\nu}_i \gg k_{\mathrm{B}}T/(hc)$, then the effective Huang-Rhys factor is defined as:

$$S_{\mathrm{eff}} = \sum_i S_i. \tag{4}$$

The effective mode wavenumber is then given by:

$$\tilde{\nu}_{\text{eff}} = \frac{\sum_i S_i \tilde{\nu}_i}{S_{\text{eff}}}. \tag{5}$$

Under this model, the reverse intersystem crossing (RISC) rate is:

$$k_{\text{RISC}}^{\text{MLJ}} = \frac{2\pi}{\hbar} \frac{|H_{\text{SO}}|^2}{\sqrt{4\pi\lambda_{\text{S}} k_{\text{B}} T}} \sum_{n=0}^{\infty} \text{e}^{-S_{\text{eff}}} \frac{{S_{\text{eff}}}^n}{n!} \exp\left[-\frac{(\Delta E_{\text{ST}} + \lambda_{\text{S}} + nhc\tilde{\nu}_{\text{eff}})^2}{4\lambda_{\text{S}} k_{\text{B}} T}\right]. \tag{6}$$

As is well known, according to the Marcus model k_{RISC} is given by:

$$k_{\text{RISC}}^{\text{M}} = \frac{2\pi}{\hbar} \frac{|H_{\text{SO}}|^2}{\sqrt{4\pi\lambda k_{\text{B}} T}} \exp\left[-\frac{(\Delta E_{\text{ST}} + \lambda)^2}{4\lambda k_{\text{B}} T}\right], \tag{7}$$

where λ is the total reorganization energy.

Eq.s 6 and 7 have been used in conjunction with the same reorganization energy to compute the RISC rates of Table S3. The reorganization energy λ_{S} appearing in Table S4 was computed as the sum of λ_i for the low-frequency excluded modes, after rescaling each λ_i by a factor equal to the ratio of the total λ predicted by TDDFT calculations and the total λ predicted by the harmonic model. However, one can argue from Table S1 that this rescaling had an effect only in a few cases, specifically when the total λ inferred from the harmonic model differed significantly from the TDDFT-predicted value. Table S4 presents the parameters (H_{SO}, S_{eff}, $\tilde{\nu}_{\text{eff}}$, and λ_{S}) used, in conjunction with ΔE_{ST} from Table S1, to compute k_{RISC} with the MLJ approach, denoted as $k_{\text{MLJ}}^{\lambda_{\text{S}}}$. Table S4 also reports λ_{fit}, which refers to the reorganization energy obtained through a fitting procedure. This procedure identifies the optimal λ_{S} to be used in the MLJ equation while keeping the other parameters (T, H_{SO}, ΔE_{ST}, $\tilde{\nu}_{\text{eff}}$, S_{eff}) fixed, so that the equation fits either the QM rate or the observed rate. In all cases, the fit provides a near exact match between the QM and MLJ rate.

Table S3: RISC rates (s^{-1}) calculated at $T = 300$ K by using the Marcus (M) and Marcus Levich Jortner (MLJ) equations in conjunction with the same value of reorganization energy (λ, eV). Three values of λ have been used: 0.04, 0.1, and 0.2 eV. Singlet-triplet energy gaps are taken from Table S1. Spin-orbit couplings are taken from Table S4. For MLJ computations, Huang-Rhys factors and wavenumbers of the effective mode are taken from Table S4.

	$\lambda = 0.04$		$\lambda = 0.1$		$\lambda = 0.2$	
Molecule ID	$k_{\mathrm{RISC}}^{\mathrm{M}}$	$k_{\mathrm{RISC}}^{\mathrm{MLJ}}$	$k_{\mathrm{RISC}}^{\mathrm{M}}$	$k_{\mathrm{RISC}}^{\mathrm{MLJ}}$	$k_{\mathrm{RISC}}^{\mathrm{M}}$	$k_{\mathrm{RISC}}^{\mathrm{MLJ}}$
PXZ-TRZ	1.03×10^{5}	9.40×10^{4}	6.16×10^{4}	5.61×10^{4}	1.97×10^{4}	1.80×10^{4}
4CzIPN	1.63×10^{6}	9.68×10^{5}	2.47×10^{6}	1.47×10^{6}	1.07×10^{6}	6.50×10^{5}
ACRFLCN	2.66×10^{7}	2.56×10^{7}	1.07×10^{7}	1.04×10^{7}	3.01×10^{6}	2.91×10^{6}
DMAC-TRZ	2.35×10^{7}	2.04×10^{7}	1.20×10^{7}	1.05×10^{7}	3.63×10^{6}	3.23×10^{6}
DMOC-DPS	1.09	7.46×10^{-1}	1.64×10^{3}	1.12×10^{3}	7.15×10^{3}	4.95×10^{3}
DPA-DPS	6.72×10^{-26}	3.49×10^{-26}	2.58×10^{-9}	1.34×10^{-9}	3.30×10^{-4}	1.71×10^{-4}
2CzPN	5.34×10^{-5}	4.37×10^{-5}	2.15×10	1.75×10	6.01×10^{2}	4.91×10^{2}
SPIRO-CN	1.84×10^{5}	1.81×10^{5}	1.10×10^{5}	1.08×10^{5}	3.51×10^{4}	3.46×10^{4}
TCz-TRZ	3.24×10^{5}	2.25×10^{5}	9.25×10^{5}	6.43×10^{5}	4.99×10^{5}	3.49×10^{5}

Table S4: Spin-orbit coupling ($|H_{\mathrm{SO}}|$, cm^{-1}) evaluated at the M06-2X/6-31G** level. Huang-Rhys factors (S_{eff}) and effective mode wavenumbers ($\tilde{\nu}_{\mathrm{eff}}$, cm^{-1}) computed according to Eq.s 4 and 5. λ_{S} (eV) evaluated as the sum of λ_i over the low-frequency modes. $k_{\mathrm{MLJ}}^{\lambda_{\mathrm{S}}}$ is the RISC rate ($T = 300$ K) predicted by using Eq. 6 and λ_{S}. For each molecule, λ_{fit} (eV) was obtained through a fitting procedure that matches k_{MLJ} with the corresponding QM-predicted or observed rate (where experimental value is available, see Table S1).

Molecule ID	$\lvert H_{\mathrm{SO}}\rvert$	S_{eff}	$\tilde{\nu}_{\mathrm{eff}}$	λ_{S}	λ_{fit}	$k_{\mathrm{MLJ}}^{\lambda_{\mathrm{S}}}$
PXZ-TRZ	0.03	0.09	926.59	0.002	0.074	1.46×10^{-2}
4CzIPN	0.38	0.52	786.82	0.028	0.078	4.60×10^{5}
ACRFLCN	0.26	0.03	676.46	0.001	0.042	8.39×10^{4}
DMAC-TRZ	0.36	0.15	429.36	0.024	0.092	2.05×10^{7}
DMOC-DPS	0.38	0.38	602.75	0.041	0.665	8.76×10^{-2}
DPA-DPS	0.21	0.66	835.02	0.029	0.199	8.64×10^{-37}
2CzPN	0.55	0.20	1045.60	0.180	0.285	3.74×10^{2}
SPIRO-CN	0.04	0.02	539.40	0.001	0.089	2.13×10^{-6}
TCz-TRZ	0.35	0.36	866.31	0.041	0.246	2.35×10^{5}
WAJPEC	0.50	0.12	409.80	0.017	0.161	2.79×10^{-60}
CAGZES	0.54	0.63	1746.90	0.040	0.889	2.96×10^{-24}
DELJIQ	0.46	0.17	850.24	0.171	0.762	6.62×10^{2}
FECGOL	0.32	0.35	649.87	0.042	0.110	8.56×10^{-10}
MUKJOR	0.16	0.64	1228.20	0.079	0.827	2.28×10^{-17}
ZINC000005389834	0.43	0.16	615.88	0.009	0.109	4.13×10^{-57}
ZINC000071814521	10.37	0.33	1052.30	0.416	0.542	1.03×10^{6}
ZERJEL02	2.29	0.11	1133.20	0.266	0.908	4.39×10^{-6}

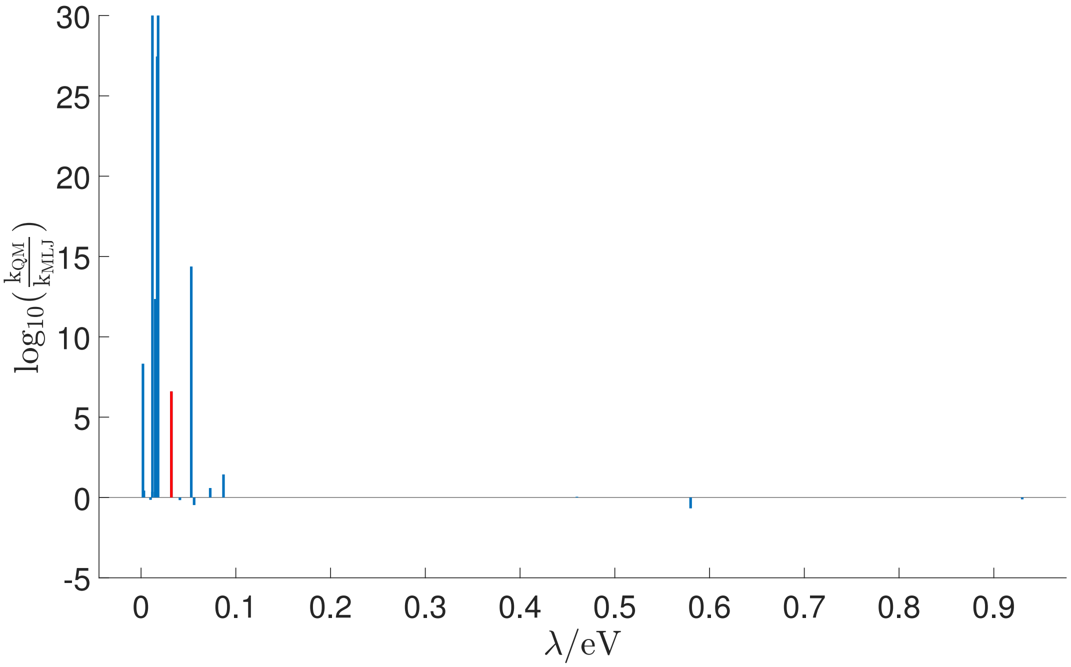

Figure S11: Decimal logarithm of the ratio between the RISC rates predicted by using the quantum mechanical method (QM) and the semi classical method (MLJ) as a function of the reorganization energy. Rates have been computed at $T = 300$ K using actual energy gaps, reorganization energies and spin orbit couplings. As expected, the biggest discrepancies between the methods are found at lower values of the reorganization energy ($\lambda < 0.1$ eV). Logarithm ratio has been cut at 30, the highest value amounts to 56. Red bar denotes the median value of λ.

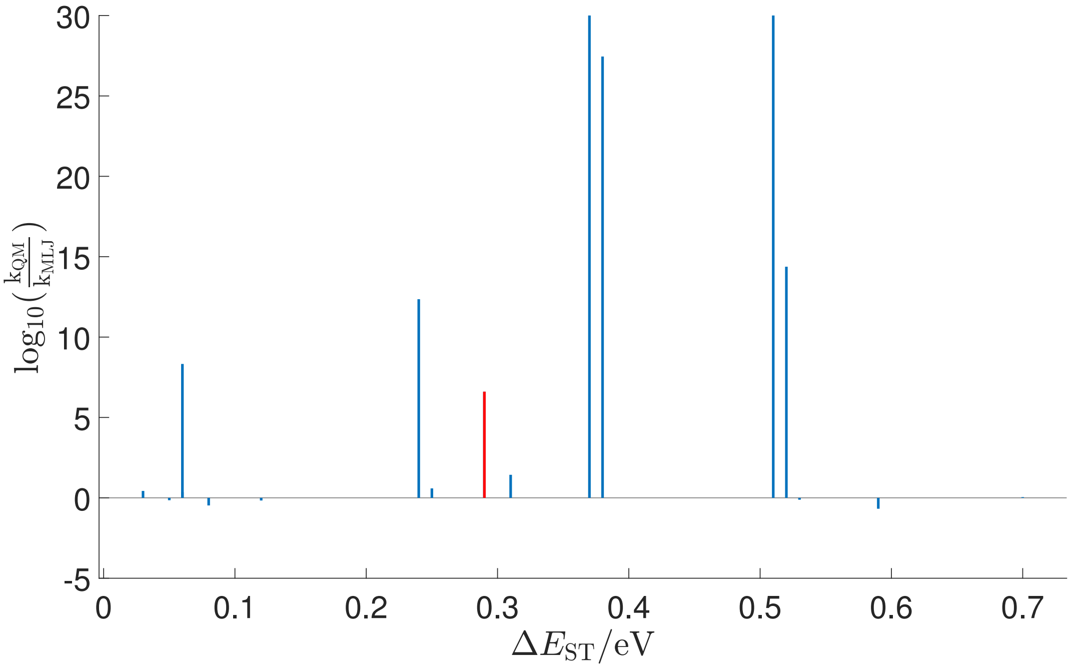

Figure S12: Decimal logarithm of the ratio between the RISC rates predicted by using the quantum mechanical method (QM) and the semi classical method (MLJ) as a function of the energy gap between S_1 and T_1. Rates have been computed at $T = 300$ K using actual energy gaps, reorganization energies and spin orbit couplings. As expected, the discrepancy between the different approaches generally increases at the increase of the energy gap. Logarithm ratio has been cut at 30, the highest value amounts to 56. Red bar denotes the median value of ΔE_{ST}.

References

(S1) Capobianco, A.; Borrelli, R.; Noce, C.; Peluso, A. Franck-Condon Factors in Curvilinear Coordinates: The Photoelectron Spectrum of Ammonia. *Theor. Chem. Acc.* **2012**, *131*, 1181.

(S2) Baiardi, A.; Bloino, J.; Barone, V. General Formulation of Vibronic Spectroscopy in Internal Coordinates. *J. Chem. Phys.* **2016**, *144*, 084114.

(S3) Fortino, M.; Bloino, J.; Collini, E.; Bolzonello, L.; Trapani, M.; Faglioni, F.; Pedone, A. On The Simulation of Vibrationally Resolved Electronic Spectra of Medium-Size Molecules: The Case of Styryl Substituted BODIPYs. *Phys. Chem. Chem. Phys.* **2019**, *21*, 3512–3526.

(S4) Samanta, P. K.; Kim, D.; Coropceanu, V.; Brédas, J.-L. Up-Conversion Intersystem Crossing Rates in Organic Emitters for Thermally Activated Delayed Fluorescence: Impact of the Nature of Singlet vs Triplet Excited States. *J. Am. Chem. Soc.* **2017**, *139*, 4042–4051.

(S5) Méhes, G.; Nomura, H.; Zhang, Q.; Nakagawa, T.; Adachi, C. Enhanced Electroluminescence Efficiency in a Spiro-Acridine Derivative through Thermally Activated Delayed Fluorescence. *Angew. Chem. Int. Ed.* **2012**, *51*, 11311–11315.

(S6) Huang, S.; Zhang, Q.; Shiota, Y.; Nakagawa, T.; Kuwabara, K.; Yoshizawa, K.; Adachi, C. Computational Prediction for Singlet- and Triplet-Transition Energies of Charge-Transfer Compounds. *J. Chem. Theory Comput.* **2013**, *9*, 3872–3877.

(S7) Tsai, W.-L.; Huang, M.-H.; Lee, W.-K.; Hsu, Y.-J.; Pan, K.-C.; Huang, Y.-H.; Ting, H.-C.; Sarma, M.; Ho, Y.-Y.; Hu, H.-C.; Chen, C.-C.; Lee, M.-T.; Wong, K.-T.; Wu, C.-C. A Versatile Thermally Activated Delayed Fluorescence Emitter for both Highly Efficient Doped and Non-Doped Organic Light Emitting Devices. *Chem. Commun.* **2015**, *51*, 13662–13665.

(S8) Wu, S.; Aonuma, M.; Zhang, Q.; Huang, S.; Nakagawa, T.; Kuwabara, K.; Adachi, C. High-Efficiency Deep-Blue Organic Light-Emitting Diodes Based on a Thermally Activated Delayed Fluorescence Emitter. *J. Mater. Chem. C* **2014**, *2*, 421–424.

(S9) Kim, G. H.; Lampande, R.; Im, J. B.; Lee, J. M.; Lee, J. Y.; Kwon, J. H. Controlling the Exciton Lifetime of Blue Thermally Activated Delayed Fluorescence Emitters Using a Heteroatom-Containing Pyridoindole Donor Moiety. *Mater. Horiz.* **2017**, *4*, 619–624.

(S10) Barbara, P. F.; Meyer, T. J.; Ratner, M. A. Contemporary Issues in Electron Transfer Research. *J. Phys. Chem.* **1996**, *100*, 13148–13168.
